# Predicting EGFR^L858R/T790M/C797S^ Inhibitory Effect of Osimertinib Derivatives by Mixed Kernel SVM Enhanced with CLPSO

**DOI:** 10.3390/ph18081092

**Published:** 2025-07-23

**Authors:** Shaokang Li, Wenzhe Dong, Aili Qu

**Affiliations:** 1 College of Computer Science and Technology, Qingdao University, Qingdao 266071, China; 2246282301@qdu.edu.cn (S.L.); 2021204468@qdu.edu.cn (W.D.); 2 School of Economics, Qingdao University, Qingdao 266071, China

**Keywords:** non-small cell lung cancer, EGFR inhibitors, XGBoost, support vector regression, comprehensive learning particle swarm optimizer, molecular docking

## Abstract

**Background/Objectives:** The resistance mutations EGFR^L858R/T790M/C797S^ in epidermal growth factor receptor (EGFR) are key factors in the reduced efficacy of Osimertinib. Predicting the inhibitory effects of Osimertinib derivatives against these mutations is crucial for the development of more effective inhibitors. This study aims to predict the inhibitory effects of Osimertinib derivatives against EGFR^L858R/T790M/C797S^ mutations. **Methods:** Six models were established using heuristic method (HM), random forest (RF), gene expression programming (GEP), gradient boosting decision tree (GBDT), polynomial kernel function support vector machine (SVM), and mixed kernel function SVM (MIX-SVM). The descriptors for these models were selected by the heuristic method or XGBoost. Comprehensive learning particle swarm optimizer was adopted to optimize hyperparameters. Additionally, the internal and external validation were performed by leave-one-out cross-validation ($Q_{LOO}^{2}$), 5-fold cross validation ($Q_{5-fold}^{2}$) and concordance correlation coefficient (CCC), $Q_{F1}^{2}$, and $Q_{F2}^{2}$. The properties of novel EGFR inhibitors were explored through molecular docking analysis. **Results:** The model established by MIX-SVM whose kernel function is a convex combination of three regular kernel functions is best: $R^{2}$ and RMSE for training set and test set are 0.9445, 0.1659 and 0.9490, 0.1814, respectively; $Q_{LOO}^{2}$, $Q_{5-fold}^{2}$, CCC, $Q_{F1}^{2}$, and $Q_{F2}^{2}$ are 0.9107, 0.8621, 0.9835, 0.9689, and 0.9680. Based on these results, the IC_50_ values of 162 newly designed compounds were predicted using the HM model, and the top four candidates with the most favorable physicochemical properties were subsequently validated through PEA. **Conclusions:** The MIX-SVM method will provide useful guidance for the design and screening of novel EGFR^L858R/T790M/C797S^ inhibitors.

## 1. Introduction

Lung cancer has long been the leading cause of cancer-related deaths worldwide, with an estimated 1.8 million deaths each year [1]. Moreover, its morbidity is so high that about 2.3 million new cases are diagnosed each year. Non-small cell lung cancer (NSCLC) accounts for almost 85% of these diagnoses. Unfortunately, since mild early symptoms tend to result in delayed diagnosis, lung cancer is often diagnosed at advanced stages when there are few therapeutic alternatives [2].

Major carcinogenic factors of NSCLC are epidermal growth factor receptor (EGFR) activating mutations, which can be effectively treated with EGFR tyrosine kinase inhibitors (TKIs) [3]. First-generation TKIs target mutated EGFR with point mutations in exon 21 (L858R) and exon 19 deletion, while second-generation TKIs work by covalently interacting with the cysteine residues (Cys797) in EGFR [4,5,6,7]. However, the T790M gatekeeper mutation, which causes acquired resistance to first-generation TKIs, is immune to second-generation inhibitors [8,9]. Osimertinib, a third-generation EGFR TKI designed to overcome the T790M mutation, has been approved by the FDA and EU due to its excellent performance in clinical trials [10]. Nevertheless, a tertiary mutation in EGFR C797S caused inevitable Osimertinib resistance, which significantly reduced the therapeutic effect during treatment of NSCLC and resulted in the requirement of developing novel inhibitors that target EGFR^C797S^ [11].

EGFR^L858R/T790M/C797S^ represents a triple mutation combination that is a major cause of acquired resistance to Osimertinib, the current third-generation EGFR tyrosine kinase inhibitor (TKI) used to treat non-small cell lung cancer (NSCLC) patients harboring the T790M mutation. While Osimertinib was specifically developed to overcome the T790M resistance mutation, the subsequent emergence of the C797S mutation severely limits its efficacy and poses a considerable challenge in clinical oncology.

Given the lack of effective therapies against this tertiary mutation, there is an urgent need to identify novel inhibitors that are effective against EGFR^L858R/T790M/C797S^. As such, we focused our modeling efforts on this triple-mutant EGFR variant to support the rational design and virtual screening of fourth-generation inhibitors that can address this critical resistance mechanism.

Previous research discovered a set of novel inhibitors, and their half maximal inhibitory concentration (IC_50_) values were measured to evaluate their inhibitory activity against EGFR^L858R/T790M/C797S^ [12,13]. Since traditional experimental assessment of inhibitory activity is costly and inefficient, a novel method is urgently needed to accelerate the development of new drugs. Quantitative structure-activity relationship (QSAR) is a computational molecular modeling methodology that utilizes mathematical and statistical methods to explore the relationship between chemical structures and biological activities or properties of compounds [14]. On the basis of carefully selected descriptors, several economical and time-efficient QSAR prediction models could be established to assist in drug design.

In this study, the linear QSAR model was established based on descriptors selected by the heuristic method (HM) while the nonlinear QSAR models were established based on descriptors selected by extreme gradient boosting (XGBoost). Since nonlinear models constructed by random forest (RF), gene expression programming (GEP), gradient boosting decision tree (GBDT), polynomial kernel function support vector machine (Poly-SVM), and mixed kernel function support vector machine (MIX-SVM) have numerous complex hyperparameters to set, the comprehensive learning particle swarm optimizer (CLPSO) was applied for hyperparameter optimization, and the dataset was randomly split multiple times to ensure the stability and consistency of the selected parameters. The prediction and validation results indicate that the MIX-SVM model, whose kernel is a convex combination of trigonometric kernel function, polynomial kernel function, and linear kernel function, is robust and powerful in prediction. Furthermore, the applicability domain of the MIX-SVM model was defined to ensure the reliability of predictions. Unlike previous studies that have applied MIX-SVM- or PSO-based models to other biological targets, this study focuses for the first time on the challenging EGFR^L858R/T790M/C797S^ mutations, using MIX-SVM enhanced by CLPSO to optimize predictive performance [3,4,6,7]. The design and property prediction of the new EGFR inhibitors were carried out, as well as the molecular docking of the best-performing compounds. In general, this study can provide useful guidance for the development of novel EGFR inhibitors.

## 2. Results

### 2.1. Results of HM

**Figure 1:**
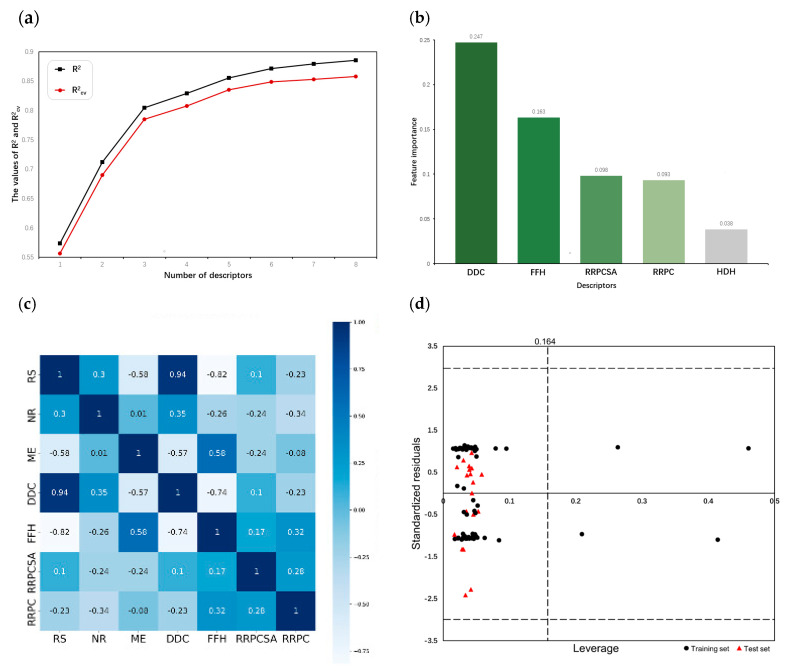
(**a**) Influence of descriptor numbers on $R^{2}$ and $R_{cv}^{2}$. (**b**) Feature importance ranking of descriptors selected by XGBoost. (**c**) Correlation heatmap of descriptors. (**d**) Williams plot of standardized residual versus leverage.

607 descriptors were calculated by CODESSA. By gradually increasing the number of descriptors from one to eight, a set of linear models were established by HM. The recorded $R^{2}$ and $R_{cv}^{2}$ demonstrate the influence of the number of descriptors on the performance of linear models. As illustrated in Figure 1a, both $R^{2}$ and $R_{cv}^{2}$ no longer increase sharply with the addition of descriptors once the number of descriptors exceeds three. Consequently, three descriptors were selected and their physical–chemical meanings are shown in Table 1.

For the training set, $R^{2}$ = 0.8094, RMSE = 0.5497; for the test set, $R^{2}$ = 0.7892, RMSE = 0.6215. Besides, $Q_{LOO}^{2}$, $Q_{5-fold}^{2}$, CCC, $Q_{F1}^{2}$, and $Q_{F2}^{2}$ are 0.7848, 0.6973, 0.8693, 0.7930, and 0.7905, respectively. The mathematical expression of the HM model is given in Equation (1):

(1) $$\mathrm{lg}\left({IC}_{50}\right)=-7.3731-40.041\times RS+0.48875\times NR+0.80855\times ME$$

### 2.2. Descriptors Selected by XGBoost

After removing all non-generic descriptors, XGBoost calculated the feature importance values of the remaining descriptors and ranked them in a non-incremental order. Figure 1b presents the top five descriptors and their corresponding feature importance. The obvious gap between the fourth and fifth descriptors in feature importance values suggests that the fifth descriptor is less relevant to the activities of the compounds. Moreover, the addition of the fifth descriptor may not significantly enhance the model but increases the risk of overfitting, which reduces the prediction performance of the model on unseen datasets [15]. Finally, four descriptors were chosen as the basis for building nonlinear models; their physical-– meanings are shown in Table 2.

To further validate the selected descriptors, SHAP (SHapley Additive exPlanations) analysis was applied to the XGBoost model. SHAP values offer consistent and locally accurate estimates of feature importance by quantifying each feature’s contribution to individual predictions.

**Figure 2:**
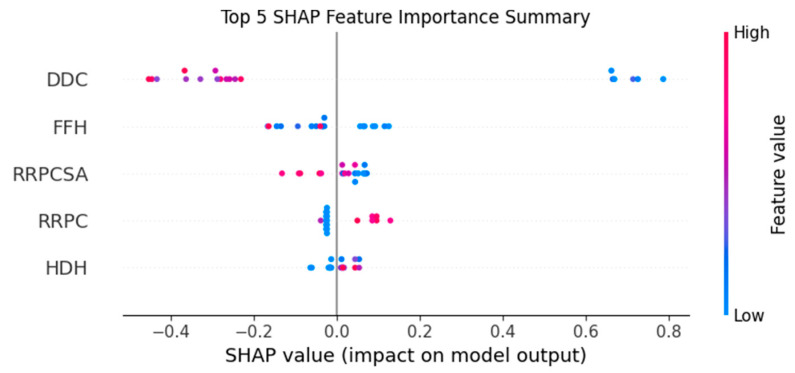
SHAP plot of descriptor importance in the XGBoost model.

Figure 2 shows that the four descriptors chosen by XGBoost have the highest SHAP values, confirming their relevance and alignment with the feature importance ranking.

This interpretability approach supports the robustness of the feature selection process and highlights the meaningful contribution of each descriptor to model performance.

Although the descriptors selected by HM and XGBoost do not overlap, the correlation coefficients between two sets of descriptors shown in Figure 1c indicate that RS is strongly correlated with DDC and FFH. Additionally, the correlation coefficient between any two descriptors selected by XGBoost is less than 0.8, confirming the validity of the descriptors selected by XGBoost.

### 2.3. Results of RF

Based on descriptors nonlinearly selected by XGBoost, the RF model was constructed, and its four key parameters were optimized by CLPSO. The RF model produced relatively good results when the parameters including the number of trees, the maximum depth of the tree, the minimum samples per split, and the minimum samples per leaf were set at 575, 6, 3, and 3, respectively. In this case, $R^{2}$ for the training set and test set are 0.8940 and 0.8779, and their corresponding RMSEs are 0.2188 and 0.3163. Additionally, $Q_{LOO}^{2}$, $Q_{5-fold}^{2}$, CCC, $Q_{F1}^{2}$, and $Q_{F2}^{2}$ are 0.8428, 0.7163, 0.9315, 0.8778, and 0.8777, respectively.

### 2.4. Results of GEP

The descriptors of selected by XGBoost were imported into APS to build the GEP nonlinear model. $R^{2}$ for the training set and test set are 0.7631 and 0.7036, and their corresponding RMSEs are 0.3812 and 0.4546. Additionally, $Q_{LOO}^{2}$, $Q_{5-fold}^{2}$, CCC, $Q_{F1}^{2}$, and $Q_{F2}^{2}$ are 0.5023, 0.4107, 0.8261, 0.7698, and 0.7701, respectively. The nonlinear model established by GEP is expressed as Equation (2):

(2) $$\begin{array}{ll} \mathrm{lg}\left({IC}_{50}\right)= & \sin\left(abs\left(\exp\left(\sin\left(\exp\left(mod\left(\cos\left(floor\left(d_{1},d_{2}\right)\right)\right)\right)\right)\right)\right)\right)\\ & +\cos\left(mod\left(abs\left(d_{1}\right),\cos\left(pow{\left(d_{2},d_{3}\right)}^{d1}\right)\right)\right)\times pow\left(d_{2},d_{3}\right)\times sqrt\left(d_{0}\right)\\ & +cos(abs(cos(d_{2}\times sin(ceil(d_{3}))))))\times floor(d_{1})\\ & +d_{1}\times exp(d_{1})\times pow(d_{0},d_{1})\times sqrt(d_{2})+sin(sin(sin(d_{1})))-d_{2}\\ & -d_{3}\times d_{1} \end{array}$$

### 2.5. Results of GBDT

The four descriptors selected by XGBoost were used to establish the GBDT nonlinear model. After CLPSO optimization, when the m = 11, the $\theta_{m}$ = 0.08, and the maximum depth of the tree is 8, the best effect of the model is obtained. $R^{2}$ for the training set and test set are 0.7780 and 0.6478, and their corresponding RMSEs are 0.3319 and 0.4769. Additionally, $Q_{LOO}^{2}$, $Q_{5-fold}^{2}$, CCC, $Q_{F1}^{2}$, and $Q_{F2}^{2}$ are 0.7091, 0.6384, 0.9126, 0.9314, and 0.9278, respectively.

### 2.6. Results of Poly-SVM

The establishment of the Poly-SVM model involves the setting of many parameters that determine its performance. Specifically, C relates to the regularization degree; if C is too large, the model tends to overfit, while if it is too small, the model tends to underfit. ε is employed to fit the training data by adjusting the ε-insensitive zone’s width. d determines the complexity of the decision boundary. Higher values of d enable the SVM to capture more intricate relationships in the data, but may also increase the risk of overfitting. γ affects the shape of the decision boundary. The model may overfit if γ is set too high, and it may underfit if γ is set too low. r balances the influence of the constant term in the polynomial kernel function.

After CLPSO optimization, the values of C, ε, d, γ, and r are 4003.28, 0.36, 3, 0.57, and 4.47, respectively. $R^{2}$ = 0.8242, RMSE = 0.3026 for the training set and $R^{2}$ = 0.8956, RMSE = 0.2240 for the test set. In addition, $Q_{LOO}^{2}$, $Q_{5-fold}^{2}$, CCC, $Q_{F1}^{2}$, and $Q_{F2}^{2}$ are 0.8417, 0.8092, 0.9555, 0.9085, and 0.9049, respectively.

### 2.7. Results of MIX-SVM

As shown in Equation (11), aside from the parameters contained in the polynomial and trigonometric kernel functions, the mixed kernel function has three weight coefficients that balance learning and generalization abilities by convex combination. An efficient parameter optimization tool is necessary since the interdependence of multiple parameters increases the difficulty and complexity of parameter adjustment.

After optimizing parameters by CLPSO, the optimal C = 1.06, ε = 0.01, σ = 800.13, γ = 98.69, r = 0.01, α = 0.83, β = 0.12. At this point, $R^{2}$ for the training set and test set are 0.9445 and 0.9490, and their RMSEs are 0.1659 and 0.1814. Additionally, $Q_{LOO}^{2}$, $Q_{5-fold}^{2}$, CCC, $Q_{F1}^{2}$, and $Q_{F2}^{2}$ are 0.9107, 0.8621, 0.9835, 0.9689, and 0.9680, respectively.

### 2.8. Design of New EGFR Inhibitors

The determinants of EGFR inhibitor IC_50_ values were examined through the analysis of molecular descriptors employed in the HM model. As shown in Table 1, the raw (non-standardized) regression coefficients represent the rate of change in IC_50_ corresponding to unit changes in each descriptor. These coefficients illustrate the individual contribution of each variable to the predicted outcome. Based on their relative influence, the five descriptors were prioritized in the following order:

RS > NR > ME.

1. “RS” denotes the relative amount of S in the compound. Increasing its value significantly decreases the value of IC_50_ [16].
2. “NR” indicates the number of rings in the compound, and its coefficient indicates that decreasing its value slightly lowers the value of IC_50_ [17].
3. “ME” represents the maximum exchange energy of the C-N bond in the compound. Lowering its value slightly reduces the value of the IC_50_ [16].

**Figure 3:**
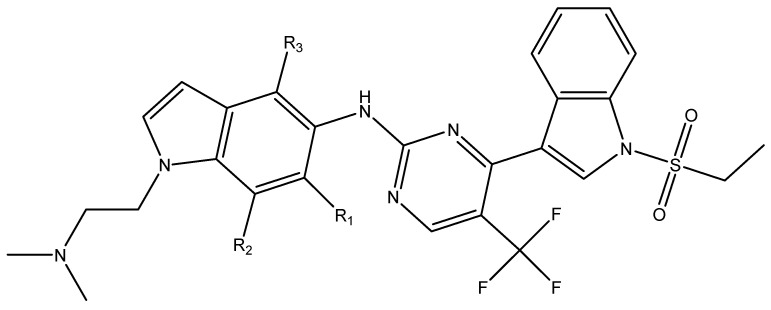
The design approach targeted the R region of compound **45**.

In conclusion, HM model and analysis of molecular descriptors have identified several key factors that influence compound activity. Compound **45** is the most potent compound reported in the literature, as it exhibits the lowest IC_50_ value. Its structural composition can be modified based on these factors. To guide the design of novel and effective EGFR inhibitors, increasing the sulfur content may contribute to improved inhibitory potency. Figure 3 illustrates the primary positions where the molecular structure has been adjusted.

To reduce the polar interactions between atoms and enhance the distribution of different charges, functional groups such as halogens, mercaptans, thioethers, and sulfones are introduced at positions R1 to R3 and combined in a random manner. Using the descriptor analysis from the HM model, a total of 162 molecules were designed.

The IC_50_ values of the compounds were predicted using the HM model. If the predicted IC_50_ of a compound is lower than that of compound **45**, it is selected for further evaluation and docking through the Property Explorer Applet (PEA) (https://www.organic-chemistry.org accessed on 10 July 2025). Ultimately, the IC_50_ values for four compounds were found to be lower than that of compound **71**. Table 3 presents the predicted IC_50_ values and total docking scores for the newly designed EGFR inhibitors.

### 2.9. Property Prediction and Molecular Docking of New EGFR Inhibitors

PEA was used to assess the properties of the new compounds. The applet provides real-time predictions of physicochemical characteristics and evaluates potential toxicity risks for user-defined chemical structures. It analyzes various compound properties, including partition coefficient, water solubility, topological polar surface area (TPSA), drug-likeness, and others.

The partition factor P represents the ratio of solute concentrations between two solvents, and LogP is the logarithm of this ratio. LogP indicates the ratio of a compound’s solubility in n-octanol to water and is a key measure of hydrophilicity. Compounds with LogP values between –2.0 and 5.0 tend to exhibit favorable absorption properties [18].

Water solubility is vital for determining the intestinal uptake and cellular distribution of compounds. Increased solubility in water typically results in better absorption of the designed molecules [19].

TPSA is the total surface area of polar atoms in a compound [20]. A higher TPSA reduces the chances of molecules crossing the membrane. Drug-likeness evaluates bioavailability potential, while the drug score combines these factors into one value, serving as a key measure of a compound’s drug candidate potential. Table 4 shows the calculated properties of the newly designed compounds from PEA and their predicted lg(IC_50_) values from the HM model.

**Figure 4:**
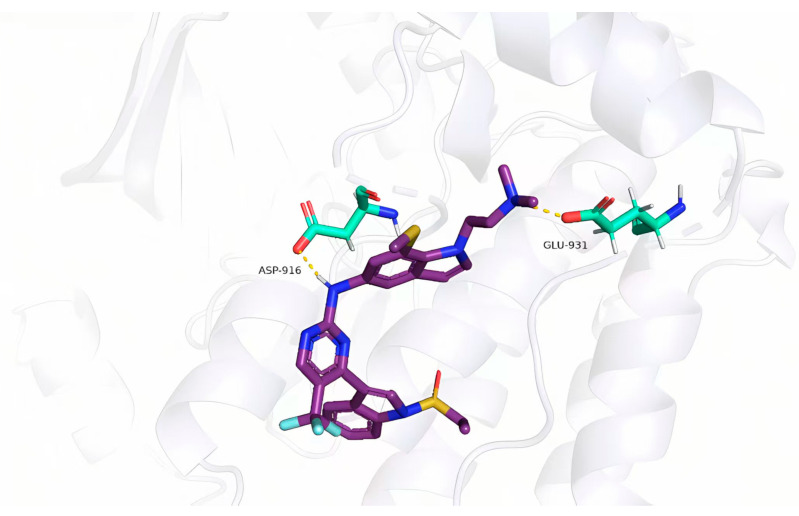
Docking analysis of compound **45c** to target protein (PDB ID: 7zyn).

In the molecular docking analysis, the newly designed compounds were assessed as potential ligands for their interactions with the target protein (PDB ID: 7zyn). Among them, compound **45c** showed the strongest binding affinity, with a total docking score of 5.0483—significantly higher than that of compound **45**. This suggests a more stable and stronger interaction between compound **45c** and the protein’s binding site. As shown in Figure 4, compound **45c** forms two key hydrogen bonds with specific residues.

The predicted docking conformation of compound **45c** revealed several key hydrogen bonds between the ligand and active site residues of the target protein. Notably, oxygen atoms in the core structure of **45c** form hydrogen bonds with GLU-931 and ASP-916, contributing to a more stable and favorable binding pose. This strong molecular interaction suggests that compound **45c** holds promise as a potential lead inhibitor of this protease.

### 2.10. Applicability Domain Analysis

The Williams plot was used to visualize the AD of the MIX-SVM model in this study. The vertical line represents the warning leverage $h^{*}$ while the horizontal lines represent the standardized residual threshold of ±3σ. When applying a QSAR model, only predicted data for chemicals within the chemical domain of the training set should be considered. In the case where the compound is outside the AD, the prediction result must be used with great care since it can be extrapolated from the model [21]. However, there is an exception. If a compound from the training set has a higher leverage value than $h^{*}$ and a standardized residual within ±3σ, it is conducive to reinforcing the model and will be considered a structurally influential chemical [22]. Figure 1d illustrates that all compounds in the test set fell within the AD and four compounds in the training set were identified as influential chemicals. This demonstrated the reliability of the MIX-SVM model’s predictions.

## 3. Discussion

**Figure 5:**
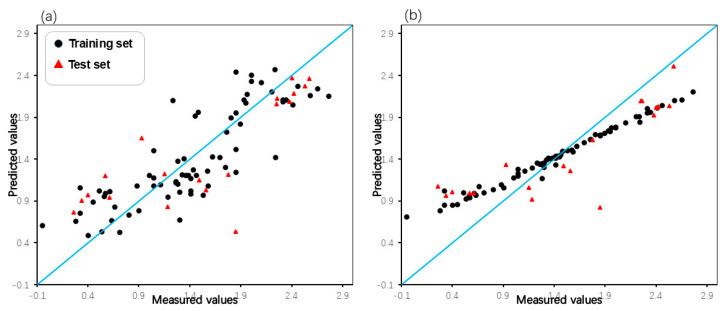
Plot of measured and predicted lg (IC_50_) of the GEP model (**a**), GBDT model (**b**).

**Figure 6:**
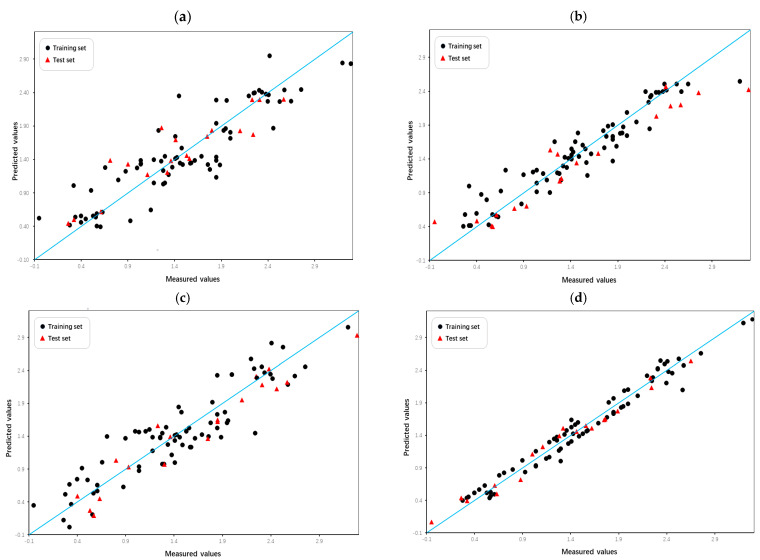
Plot of measured and predicted lg (IC_50_) of the HM model (**a**), RF model (**b**), Poly-SVM model (**c**), and MIX-SVM model (**d**).

Intuitive comparison of the prediction results is shown in Figure 5 and Figure 6. Due to the limitations of linear methods, the HM model did not produce satisfactory results. Overfitting has occurred with the GEP and GBDT models, which have average fitting effects. In contrast, the RF model not only avoided overfitting but also achieved high prediction scores, demonstrating its excellence in the domain of machine learning applications. In terms of models built by SVM, the Poly-SVM model failed to achieve high performance on the training set due to limited learning capability, and its leave-one-out cross-validation result was also affected. Benefitting from the complementary effect of the convex combination, the mixed kernel function reinforces its learning and generalization abilities by combining the advantages of the trigonometric, polynomial, and linear kernel functions. The results demonstrate that the MIX-SVM model fits the data best and exhibits excellent robustness. In addition, to evaluate the reproducibility of the model parameters, the dataset was randomly split multiple times, and similar performance was observed in the run. This consistency confirms the robustness of the hyperparameters chosen by CLPSO to changes in data partitioning. And the y-randomization test results shown in Table 5 confirm the absence of any chance correlation. In summary, a series of nonlinear models established on the basis of descriptors selected by XGBoost perform better. This further validates the effectiveness of XGBoost as a nonlinear feature selection technique.

## 4. Experimental Section

### 4.1. Data Preparation

All the compounds were from the literature [12,13] and their EGFR^L858R/T790M/C797S^ IC_50_ values were measured under the same experimental environment. All target compounds synthesized have a purity of over 95%. The purity and mass spectra of the compounds were analyzed by HPLC-MS (SHIMADZU LCMS-2020), and NMR spectra were run on Bruker NMR Inova 300, 400, or 600 spectrometers to ensure their purity and structural correctness. Their structures and corresponding IC_50_ values are listed in Table 6. The dataset was divided at random in a 4:1 ratio: the training set which comprised 73 compounds was used to construct models, while the remaining 19 compounds constituted the test set for evaluation.

### 4.2. Descriptor Calculation

Calculation of molecular descriptors, which is the primary task in the process of building QSAR models, was executed by the following steps: First, ChemDraw8.0 (https://revvitysignals.com accessed on 10 May 2025) acted as a tool for drawing the structures of all compounds. Second, pre-optimization in the MM+ molecular mechanical force field and more accurate optimization by the semi-empirical method were performed by HyperChem4.0 (http://hypercubeusa.com accessed on 11 May 2025) [23] according to the principle of minimum energy [24]. Then, software MOPAC6.9 (Stewart Computational Chemistry MOPAC Home Page accessed on 11 May 2025) [25] converted the results into MNO files, which were the input for CODESSA2.64 (https://compudrug.com accessed on 12 May 2025). Finally, five classes of molecular descriptors, including constitutional, electrostatic, geometrical, topological, and quantum chemical descriptors, were obtained from CODESSA [26].

### 4.3. Linear Model by HM

Descriptor pre-selection is a prerequisite for building accurate and efficient models. When two or more descriptors in a regression model are highly correlated, multicollinearity occurs, making it difficult to isolate the individual effect of each descriptor on the dependent variable. This can lead to unstable regression coefficients, reduced model interpretability, and overfitting. During this procedure, descriptors with constant values, F-test values under 1.0 in the one-parameter correlation, correlation coefficients exceeding 0.8, and t-values below the user-specified value, as well as non-universal descriptors, were discarded [27]. Then linear regression models can be built based on the rest of the descriptors. HM is a universal approach for selecting descriptors and constructing linear models, and prior research has demonstrated its effectiveness [28,29,30]. The number of descriptors included in a linear model has a significant impact on model performance; the more descriptors a model contains, the stronger its prediction ability.

### 4.4. Nonlinearly Selecting Descriptors by XGBoost

Nonlinear dimensionality reduction, which can be done by mapping a low-dimensional surface to a high-dimensional space to find nonlinear relationships among features, is conducive to improving the performance of nonlinear models [31]. To enhance the performance of the nonlinear models constructed in this study, the optimized gradient tree boosting algorithm XGBoost was applied to nonlinearly compute the feature importance of each descriptor to obtain a minimized and optimal set of descriptors. Feature importance of XGBoost could be calculated based on different metrics, and the split gain method was adopted in this study because of its advantages in capturing more subtle relationships among features [32]. By calculating the information gain when splitting the decision trees, the significance of a feature could be expressed numerically.

(3) $$w_{\sigma }^{2}\left(T\right)=\sum_{i=1}^{N}\tau_{i}^{2}$$

Equation (3) was proposed to measure the importance of each feature $X_{\sigma }$ in a single decision tree T [33]. N represents the number of internal nodes in the decision tree, and feature $X_{\sigma }$ divides the area into left and right subareas at every node i. The feature that maximizes the estimated improvement $\tau_{i}^{2}$ in the squared error risk will be chosen [34]. The squared importance of the feature $X_{\sigma }$ is the accumulation of squared improvement over the N nodes. XGBoost feature importance over M decision trees is calculated as Equation (4).

(4) $$w_{\sigma }^{2}\left(T\right)=\frac{1}{M}\sum_{m=1}^{M}\tau_{i}^{2}\left(T_{m}\right)$$

### 4.5. Nonlinear Model by RF

As one of the most popular bagging algorithms, RF is particularly adept at handling large-scale datasets with missing values and is widely used for classification and regression [35]. Based on a large number of uncorrelated decision trees, the result can be obtained by the trees’ voting for classification or averaging for regression [36]. By adopting the bagging strategy and aggregating decision trees, RF reduces the risk of overfitting and improves prediction accuracy [37].

There are four crucial parameters that should be determined in the process of building an RF model. The number of trees which controls the number of decision trees in the forest is positively correlated with the computational complexity. The maximum depth of the tree controls the depth of the tree and bigger values will lead to overfitting with higher possibility. The minimum samples per split controls the minimum number of samples required for a node split, and an excessive value may cause underfitting. The minimum samples per leaf, which is relative to the pruning, controls the minimum number of samples that a node must hold after being split.

### 4.6. Nonlinear Model by GEP

A nonlinear model was developed using gene expression programming (GEP) to predict IC_50_ of compounds, given the non-linear and complex relationship between factors affecting the ability of EGFR inhibitors [38].

The discovery of biological gene structure and function forms the basis of GEP, a new type of adaptive evolution algorithm. GEP was developed on the basis of genetic algorithms (GA) and genetic programming (GP) that absorb the advantages of both but overcome the disadvantages of both, and its distinctive feature is that it can solve complex problems with simple coding [39]. The process of GEP is as follows. GEP first decodes the problem into fixed-length chromosomes, whose head has functions and terminals and tail only has terminals. The tail length is determined by the length of the head to ensure legitimacy. Then, the population is iteratively evolved through selection, crossover, mutation, and other operations, and the individuals are retained according to the fitness assessment. Evolution leads to changes in chromosome structure, such as function combinations or terminal substitutions. Ultimately, the optimum chromosome is encoded into an expression tree and translated into a mathematical model or solution for the task.

GEP is widely used in the scientific field, as it is integrated into automatic problem solver2.9 (APS) (http://www.gepsoft.com accessed on 2 June 2025), a commercial software developed by Ferreira. The software is capable of encoding the molecular descriptors chosen by XGBoost that are most relevant to inhibitor activity, and developing nonlinear models to estimate IC_50_ values of EGFR inhibitors.

### 4.7. Nonlinear Model by GBDT

The gradient boosting decision tree (GBDT), which is an ensemble learning method, predicts target variables by constructing many decision trees in a sequential fashion. To improve accuracy, the prediction of the model is refined in every GBDT iteration. With each iteration, GBDT builds a new decision tree to reduce the total prediction error by combining predictions from previous iterations and focusing on the residuals that are the difference between projected and actual values [40]. A complex model is able to be constructed by GBDT through multiple iterations, which enables it to capture nonlinear relationships in data and make more accurate predictions. The principle of GBDT is shown in Equation (5).

(5) $$f_{m}\left(x\right)=f_{m-1}+T\left(x,\theta_{m}\right)$$

m represents m–th iterations and has m decision improvement trees. $f_{m}(x)$ is the predicted value of the model. $T\left(x,\theta_{m}\right)\;$ represents the m–th decision tree. $\theta_{m}$ is the learning rate that controls the contribution of each tree. The difference between the boosted trees of GBDT in different problems is the difference in the loss function, and the squared error loss is used in the regression problem [41]. The final optimization objective function is shown in Equation (6).

(6) $$L\left(y_{i},f_{m-1}\left(x\right)+T\left(x,\theta_{m}\right)\right)={\left(r-T\left(x,\theta_{m}\right)\right)}^{2}$$

$y_{i}$ represents the true value. r denotes the residual. The m–th decision $T(x,\theta_{m})$ tree fits the residuals.

### 4.8. Nonlinear Model by Poly-SVM

The support vector machine (SVM) proposed by Vapnik to solve the binary classification problems initially is a powerful supervised machine learning algorithm [42]. When SVM is used for regression, it is also known as support vector regression (SVR). After mapping the training data into a high-dimensional feature space, SVR aims to identify the optimal hyperplane that minimizes the training error of the data based on the ε-insensitive loss function [43]. To further regulate the SVM restrictions, slack variables $\xi_{i}$ and $\xi_{i}^{*}$ are added to deal with non-perfectly separable cases, and a penalty parameter C is introduced to control the trade-off between the margin’s size and the penalty associated with the slack variables. The final optimization objective function is shown in Equation (7).

(7) $${min}_{w{,\xi }_{i},\xi_{i}^{*}}=\frac{1}{2}{\left\|w\right\|}^{2}+C\sum_{i=1}^{n}\left(\xi_{i}+\xi_{i}^{*}\right)\;\;\;\;\;\;s.t.\;\left\{\begin{array}{c} y_{i}-w\cdot x_{i}-b\leq \varepsilon +\xi_{i},\\ w\cdot x_{i}+b-y_{i}\leq \varepsilon +\xi_{i}^{*},\\ \xi_{i}\cdot \xi_{i}^{*}\geq 0. \end{array}\right.$$

where w, x, and b represent the weight vector, input feature vector, and bias, respectively. Besides, the kernel function K is devised to transform the data into a higher-dimensional space to separate linearly inseparable data with low computational complexity [44]. After introducing the Lagrange multipliers $\alpha_{i}$ and $\alpha_{i}^{*}$, the function can be simplified as Equation (8).

(8) $$f\left(x\right)=\sum_{j=1}^{n}\left(\alpha_{j}-\alpha_{j}^{*}\right)K\left(x_{i},x_{j}\right)+b$$

Commonly used kernel functions include linear kernel function, Gaussian kernel function, polynomial kernel function, and sigmoid kernel function. In Poly-SVM, the polynomial kernel function is shown in Equation (9).

(9) $$K\left(x_{i},x_{j}\right)={\left(\gamma x_{i}^{T}x_{j}+r\right)}^{d},\;\;\gamma >0$$

where d is the degree, γ is the kernel coefficient, and r is an offset coefficient.

### 4.9. Nonlinear Model by MIX-SVM

The polynomial kernel function is a powerful global kernel function in terms of generalization ability, but its learning ability is limited. And the linear kernel function has the same limitation. On the other hand, the trigonometric function was proposed which can be expressed as Equation (10) [45].

(10) $$K\left(x_{i},x_{j}\right)=\sin\left(\frac{\pi }{2+\sigma {\left\|x_{i}-x_{j}\right\|}^{2}}\right)$$

where σ is a positive real parameter. The parameter σ directly affects the model’s performance. Small σ values are suitable for sparse datasets, while large σ values are suitable for compact datasets. Results show that models built by the trigonometric kernel function SVM have excellent learning ability but relatively poor generalization ability [45].

Considering the characteristics and complementarity of various kernel functions, a new mixed kernel function, which was a convex combination of the trigonometric kernel function, polynomial kernel function, and linear kernel function, was proposed according to Mercer’s theorem [46,47]. The expression of the mixed kernel function is expressed as Equation (11).

(11) $$K_{MIX}=\alpha \cdot K_{Trig}+\beta \cdot K_{Poly}+(1-\alpha -\beta )\cdot K_{Linear}$$

where $K_{Trig}$, $K_{Poly}$, and $K_{Linear}$ represent the trigonometric kernel function, polynomial kernel function, and linear kernel function, respectively. α and β are weight coefficients ranging from 0 to 1 used for balancing the learning capability and generalization capability of the mixed kernel function.

### 4.10. Optimization by CLPSO

As the process of building nonlinear models involves many important hyperparameters, an efficient optimization algorithm is necessary to improve the efficiency and convergence speed of hyperparameter search. Compared to traditional methods like grid search or Bayesian optimization, CLPSO offers stronger global optimization ability with fewer assumptions about the objective function. Its ability to maintain population diversity helps avoid local optima, making it well-suited for tuning multiple interdependent hyperparameters in complex nonlinear models like MIX-SVM.

The particle swarm optimizer (PSO) is a population-based optimization algorithm that simulates the behavior of animal swarms that forage in a cooperative way. In PSO, each member of the swarm is called a particle, identified by velocity V and position X, and represents a possible solution in the search space. A particle updates its position and velocity as Equations (12) and (13):

(12) $$V\leftarrow \omega \times V+c_{1}\times rand1\times \left(pbest-X\right)+c_{2}\times rand2\times \left(gbest-X\right)$$

(13) X←X+V

where pbest and gbest represent the best previous position of each particle and the best position found by the whole population, respectively. $c_{1}$ and $c_{2}$ are acceleration constants; rand1 and rand2 are random numbers from [0, 1]. The inertia weight ω can balance the local and global search abilities.

As shown in Equation (12), both pbest and gbest have an impact on the update of all particles, even though the distance between the global optima and the current gbest is large. When there are many local optimal solutions in the search space, particles can easily become stuck in local optima and fail to find the global optimum [48]. The CLPSO which adopted a better learning strategy to overcome the PSO’s drawback of converging to local optimum easily was proposed [49], and it updates the velocity as Equation (14).

(14) $$V\leftarrow \omega \times V+c\times rand\times \left({pbest}_{i}-X\right)$$

where i determines which particles’ pbests should be followed. This novel strategy uses information from all particles to update a particle’s velocity, which prevents premature convergence by preserving population diversity and makes CLPSO stand out among PSO variants [48].

### 4.11. Evaluation and Validation

The coefficient of determination ($R^{2}$) ranging from 0.0 to 1.0 is a common index used to evaluate models and its higher value indicates better prediction performance of a model. It quantifies the proportion of variance explained by a regression model, which measures the success of a model constructed with independent variables in predicting the dependent variable [50]. The root mean square error (RMSE) was also used to assess model performance. RMSE values are always non-negative; a lower bound of zero indicates a perfect fit, whereas values for underperforming models increase indefinitely [51].

Validation which tests the reliability of a model in predicting the activities of compounds is an indispensable part of QSAR model development. Rigorous validation consists of three parts: the internal validation aims to verify the reproducibility of a model, the external validation aims to verify its generalizability, and the y-randomization test aims to detect chance correlations. This study performed internal validation by $Q_{LOO}^{2}$ of leave-one-out cross-validation (LOO-CV) and 5-fold cross validation (5-fold). Additionally, statistics such as the concordance correlation coefficient (CCC), $Q_{F1}^{2}$, and $Q_{F2}^{2}$ were used in external validation to measure the generalizability of the model. Additionally, the y-randomization test was performed ten times to obtain the $R_{yrand}^{2}$ and $Q_{yrand}^{2}$ of each random model.

Based on empirical values, the acceptable thresholds for a model are: $R^{2}$ > 0.7, $Q_{LOO}^{2}$ > 0.6, $Q_{5-fold}^{2}$ > 0.55, CCC > 0.85, and $Q_{Fn}^{2}$ > 0.7 [52]. And a QSAR model without chance correlations should have low $R_{yrand}^{2}$ and $Q_{yrand}^{2}$ values.

### 4.12. Applicability Domain

As reliable predictions are mostly limited to chemicals that are structurally similar to the compounds in the training set, it is imperative to evaluate the similarity of the new molecule and limit the applicability domain (AD) of the model before applying a QSAR model to screen compounds [53]. The widely used leverage method was adopted to define AD in this study. The leverage of a compound is defined as Equation (15).

(15) $$h_{i}=x_{i}^{T}{\left(X^{T}X\right)}^{-1}x_{i}$$

where X is the descriptor matrix of the training set compounds and $x_{i}$ is the descriptor vector of the query compound. The warning leverage $h^{*}$ is defined as 3×p/n. Specifically, p is the number of model parameters and n is the number of training compounds [54]. Any compound with a greater leverage value than $h^{*}$ will be regarded as outside the AD.

### 4.13. Property Prediction and Molecular Docking

In drug discovery, achieving strong binding affinity to the target is essential for identifying potential drug candidates. Additionally, crucial drug-like properties such as pharmacokinetics and toxicity profiles play a vital role in determining whether a compound advances to clinical development. The QSAR model, built on a set of carefully selected molecular descriptors that correlate with the activity of a compound, is invaluable in this process. It aids in the analysis of data to predict the behavior of new pharmaceuticals [55].

Molecular docking is a central tool in structural molecular biology and computer-aided drug design [56]. The goal of ligand–protein docking is to predict the primary binding mode between a ligand and a known three-dimensional protein structure. In this study, Sybyl-X2.1 software (https://www.scientificcomputing.com accessed on 15 July 2025) was employed to explore the potential interactions between the newly designed EGFR inhibitors and the target protein at the binding site. PyMol3.2 software (http://www.pymol.org/pymol accessed on 15 July 2025) is used to visualize the docking results.

## 5. Conclusions

This study reveals that the nonlinearly selected descriptors, including DPSA-3 Difference in CPSAs (PPSA3-PNSA3) [Quantum-Chemical PC], FHASA Fractional HASA (HASA/TMSA) [Quantum-Chemical PC], RPCS Relative positive charged SA (SAMPOS*RPCG) [Zefirov’s PC], and RPCG Relative positive charge (QMPOS/QTPLUS) [Zefirov’s PC], have a significant impact on the IC_50_ values of the novel inhibitors against EGFR^L858R/T790M/C797S^. More importantly, the nonlinear regression model built by MIX-SVM exhibits excellent prediction performance and robustness, proving the MIX-SVM to be a promising modeling method in the research of the new EGFR^L858R/T790M/C797S^ inhibitors. In addition, four novel EGFR inhibitors were designed using the descriptors identified by the HM model, followed by property prediction and molecular docking analysis. Among them, compound **45c**, which achieved a high drug score in PEA, also showed strong performance in molecular docking with a total score of 5.0483. To sum up, this research could offer helpful guidance for the development of novel EGFR^L858R/T790M/C797S^ inhibitors and the treatment of NSCLC.

## Figures and Tables

**Table 1:** Physical–chemical meaning and coefficient of HM selected descriptors.

Physical–Chemical Meaning	Symbol	Coefficient
Relative number of S atoms	RS	−40.041
Number of rings	NR	0.48875
Max exchange energy for a C-N bond	ME	0.80855

**Table 2:** Physical–chemical meanings of descriptors selected by XGBoost.

Physical–Chemical Meaning	Symbol
DPSA-3 Difference in CPSAs (PPSA3-PNSA3) [Quantum-Chemical PC]	DDC
FHASA Fractional HASA (HASA/TMSA) [Quantum-Chemical PC]	FFH
RPCS Relative positive charged SA (SAMPOS*RPCG) [Zefirov’s PC]	RRPCSA
RPCG Relative positive charge (QMPOS/QTPLUS) [Zefirov’s PC]	RRPC
HA dependent HDSA-1 [Zefirov’s PC]	HDH

**Table 3:** Predicted lg(IC_50_) by HM and docking total score of new EGFR inhibitors.

No.	EGFR Inhibitors	Predicted lg (IC_50_)	Total Score
45	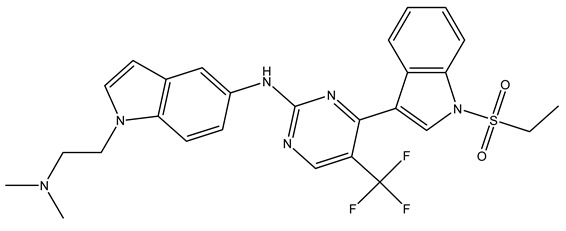	0.64	4.8087
45a	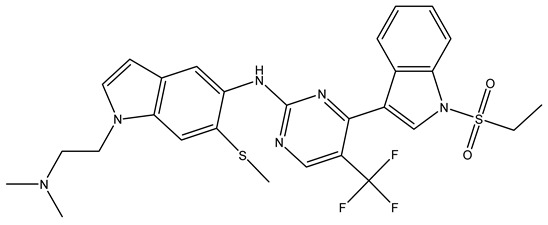	0.62	4.8125
45b	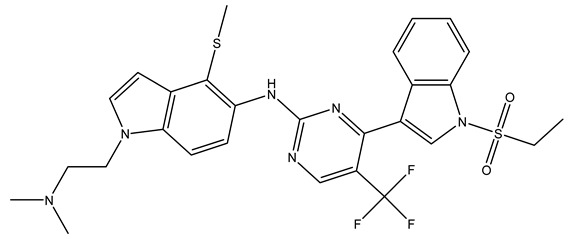	0.63	4.8103
45c	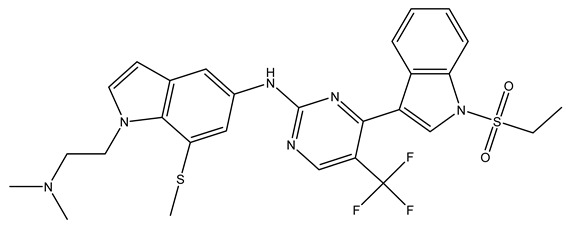	0.56	5.0483
45d	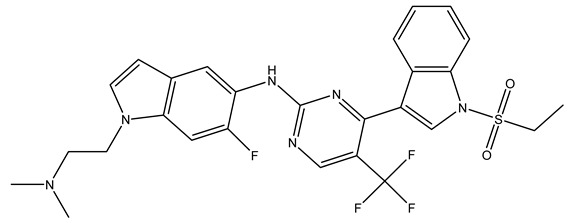	0.60	4.8394

**Table 4:** Predicted lg(IC_50_) by HM and properties by PEA of newly designed compounds.

No.	Pre.lg (IC_50_)	LogP	Solubility	Mol Weight	TPSA	Drug Likeness	Drug Score
45	0.64	3.58	−7.51	556.0	93.43	−2.85	0.17
45a	0.62	4.06	−8.36	602.0	118.7	−0.91	0.18
45b	0.63	4.06	−8.36	602.0	118.7	−0.85	0.18
45c	0.56	4.06	−8.03	618.9	116.7	−0.67	0.19
45d	0.60	3.68	−7.83	574.0	93.43	−0.91	0.20

**Table 5:** The y-randomization test results of the MIX-SVM model.

	$R_{yrand}^{2}$	$Q_{yrand}^{2}$
random 1	0.0208	−0.1850
random 2	0.0207	−0.1943
random 3	0.0016	−0.3308
random 4	0.0663	−0.1514
random 5	0.0152	−0.0859
random 6	0.0440	−0.4301
random 7	0.0003	−0.4650
random 8	0.0010	−0.3564
random 9	0.0597	−0.1834
random 10	0.0922	−0.2965

**Table 6:** Measured and predicted lg (IC_50_) of EGFR^L858R/T790M/C797S^ inhibitors.

							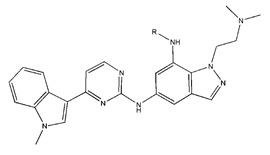							
Compound		R					Measured IC_50_ (nM)	Measured lg (IC_50_)			Predicted Ig (IC_50_)			
									HM	RF	GEP	GBDT	Poly-SVM	MIX-SVM
1 *		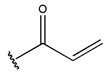					177	2.25	2.39	2.31	2.05	2.06	2.31	2.26
2 *		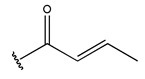					180	2.26	2.39	2.33	2.13	2.06	2.28	2.34
3		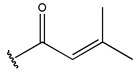					378	2.58	2.43	2.39	2.16	2.06	2.18	2.32
4		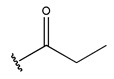					217	2.34	2.40	2.38	2.11	1.94	2.36	2.35
5 *		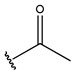					242	2.38	2.37	2.39	2.08	1.92	2.42	2.37
6		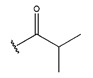					572	2.76	2.44	2.38	2.15	2.16	2.45	2.75
							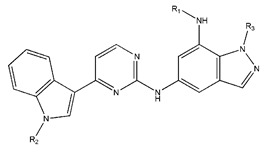							
Compound		R_1_		R_2_		R_3_	Measured IC_50_ (nM)	Measured lg (IC_50_)			Predicted Ig (IC_50_)			
									HM	RF	GEP	GBDT	Poly-SVM	MIX-SVM
7 *		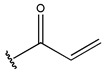		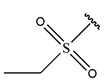		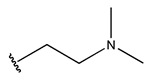	31	1.49	1.32	1.43	1.14	1.34	1.26	1.33
8		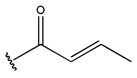		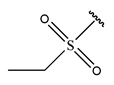		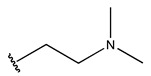	22	1.34	1.17	1.42	1.40	1.41	1.27	1.35
9		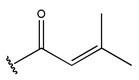		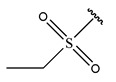		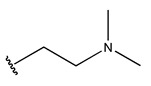	29	1.46	1.34	1.34	1.20	1.45	1.38	1.45
10		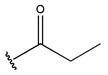		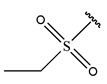		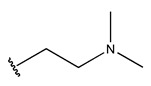	27	1.43	1.42	1.5	1.27	1.45	1.42	1.44
11		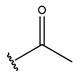		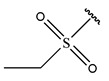		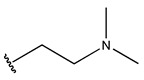	11	1.04	1.38	1.23	1.50	1.21	1.46	1.05
12		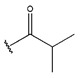		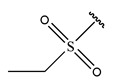		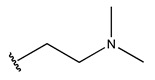	37	1.57	1.34	1.34	1.26	1.51	1.23	1.56
13		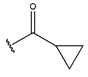		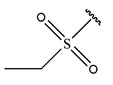		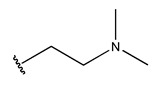	18	1.26	1.87	1.47	1.13	1.35	1.38	1.27
14		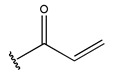		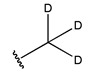		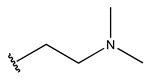	448	2.65	2.27	2.5	2.24	2.07	2.31	2.64
15 *		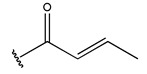		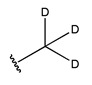		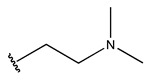	249	2.40	2.26	2.5	2.36	1.99	2.34	2.29
16		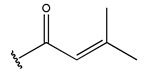		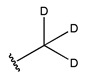		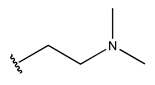	206	2.31	2.43	2.38	2.09	1.98	2.18	2.32
17		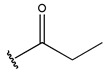		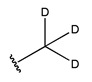		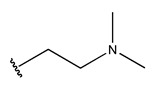	256	2.41	2.36	2.47	2.05	1.99	2.81	2.42
18		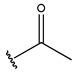		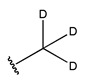		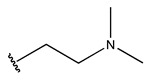	158	2.20	2.35	2.39	2.20	1.89	2.57	2.21
19 *		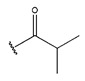		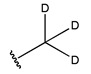		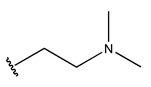	341	2.53	2.26	2.5	2.27	2.01	2.75	2.57
20 *		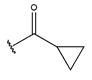		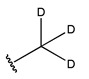		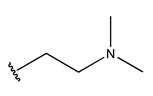	261	2.42	2.95	2.41	2.18	2.00	2.27	2.41
21		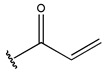		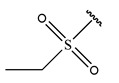		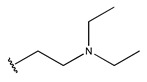	42	1.62	1.38	1.47	1.43	1.55	1.36	1.23
22		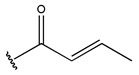		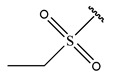		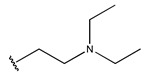	19	1.28	1.23	1.18	1.37	1.36	1.44	1.29
23		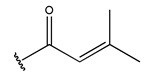		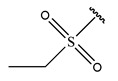		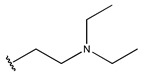	23	1.36	1.38	1.27	1.20	1.40	1.39	1.40
24		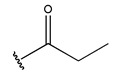		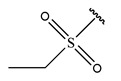		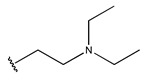	21	1.32	1.20	1.29	1.21	1.39	1.53	1.31
25		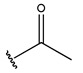		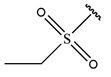		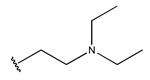	13	1.11	1.17	1.18	1.10	1.26	1.47	1.12
26 *		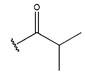		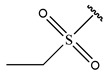		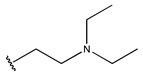	60	1.78	1.25	1.73	1.21	1.67	1.6	1.44
27		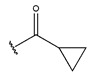		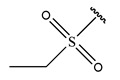		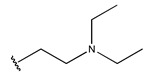	26	1.41	1.74	1.45	1.02	1.41	1.33	1.42
							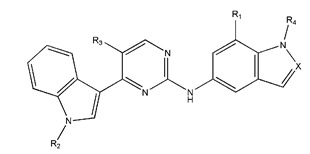							
Compound	X	R_1_	R_2_		R_3_	R_4_	Measured IC_50_ (nM)	Measured lg (IC_50_)			Predicted Ig (IC_50_)			
									HM	RF	GEP	GBDT	Poly-SVM	MIX-SVM
28 *	C	H	-CH_3_		H	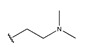	375	2.57	2.30	2.2	2.36	2.46	2.22	2.22
29	C	H	-CH_3_		H	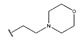	1571	3.20	2.84	2.54	2.26	2.42	3.05	3.19
30	C	CI	-CH_3_		H	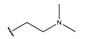	170	2.23	2.29	2.23	2.47	1.89	2.42	2.22
31	C	CI	-CH_3_		H	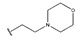	1963	3.29	2.83	2.42	2.44	2.47	2.93	3.28
32	C	H	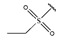		H	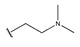	7.6	0.88	1.22	0.73	1.08	1.10	0.62	0.87
33	C	H	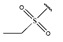		H	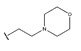	56	1.75	1.74	1.81	1.30	1.63	1.36	1.76
34	C	CI	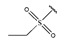		H	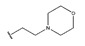	175	2.24	1.77	1.84	1.42	1.81	1.44	1.89
35	C	H	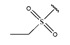		H	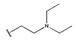	11	1.04	1.32	0.91	1.17	1.24	0.93	0.62
36	C	CI	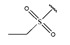		H	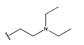	24	1.38	1.28	1.4	1.21	1.41	1.11	1.37
37	C	H	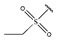		H	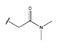	38	1.58	1.35	1.15	1.08	1.50	1.23	1.57
38	N	H	-CH_3_		CI	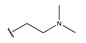	71	1.85	2.28	1.9	1.30	1.67	2.32	1.86
39	N	H	-CH_3_		-OCH_3_	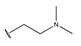	204	2.31	2.29	2.03	1.42	1.93	2.45	2.30
40	N	H	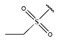		CI	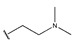	4.6	0.66	1.27	0.92	1.17	1.10	1	0.67
41	N	CI	-CH_3_		-NO_2_	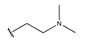	100	2.00	1.80	2.08	1.21	1.76	2.33	2.01
42	C	H	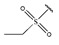		H	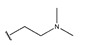	1.9	0.28	0.42	0.57	1.08	0.82	0.51	0.29
43	C	H	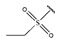		CI	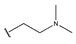	2.8	0.45	0.51	0.87	2.44	0.87	0.91	0.71
44	N	CI	-CH_3_		-CH_3_	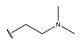	92	1.96	2.28	1.87	2.11	1.76	1.63	1.97
45 *	C	H	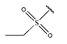		-CF_3_	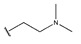	14	1.15	0.64	1.08	1.22	1.07	1.5	1.15
46	C	H	-CH_3_		-NO_2_	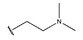	100	2.00	1.71	1.74	0.83	1.76	2.33	1.99
47	N	-NH_2_	H		H	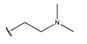	30	1.48	1.57	1.78	2.40	1.49	1.76	1.53
							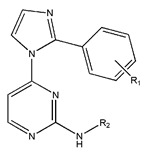							
Compound		R_1_			R_2_		Measured IC_50_ (nM)	Measured lg (IC_50_)			Predicted Ig (IC_50_)			
									HM	RF	GEP	GBDT	Poly-SVM	MIX-SVM
48		4-F			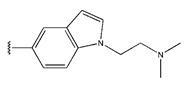		17	1.23	1.83	1.65	0.66	1.36	1.56	1.48
49		4-CI			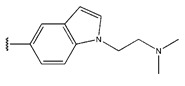		86	1.93	1.83	1.77	0.89	1.72	1.76	1.92
50		4-OMe			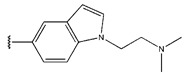		289	2.46	1.86	2.18	2.17	2.01	2.12	2.45
51		3-F			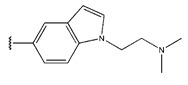		90	1.95	1.86	1.78	2.33	1.71	1.6	1.52
52		4-F			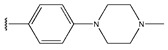		63	1.80	1.83	1.88	1.96	1.68	1.91	1.81
53		4-F			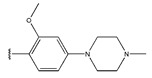		127	2.10	1.83	1.94	2.10	1.81	1.95	2.09
54		4-F			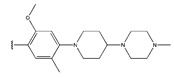		28	1.45	2.34	1.65	2.10	1.43	1.84	1.46
							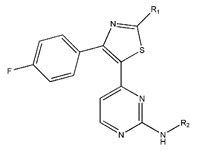							
Compound		R_1_			R_2_		Measured IC_50_ (nM)	Measured lg (IC_50_)			Predicted Ig (IC_50_)			
									HM	RF	GEP	GBDT	Poly-SVM	MIX-SVM
55		-NH_2_			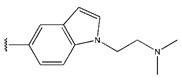		2.1	0.32	1.00	0.99	2.27	1.08	0.66	0.33
56		-NHCH_3_			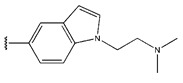		19	1.28	1.02	1.07	2.07	1.16	0.97	1.27
57		-CH_3_			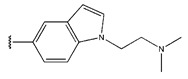		11	1.04	1.34	1.04	1.89	1.29	0.87	1.05
58		-CH(CH_3_)_2_			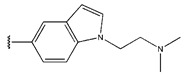		20	1.30	1.44	1.08	2.31	1.29	0.97	1.29
59		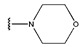			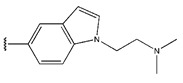		26	1.41	1.70	1.39	1.92	1.32	0.99	1.40
60		-NHCOCH_3_			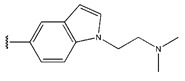		3.2	0.51	0.93	0.79	1.06	1.03	0.73	0.52
61		-NHSO_2_Me			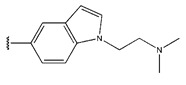		2.5	0.40	0.46	0.48	1.10	0.88	0.49	0.39
62		-NHSO_2_Et			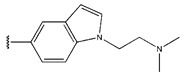		0.9	−0.05	0.52	0.47	1.08	0.74	0.34	0.18
63		-NHSO_2_iPr			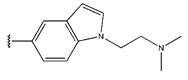		3.4	0.53	0.55	0.42	1.00	0.95	0.27	0.52
64		-NHSO_2_nBu			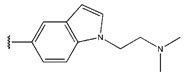		4.3	0.63	0.61	0.54	0.98	0.99	0.45	0.64
65 *		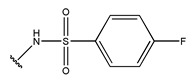			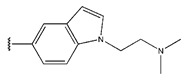		15	1.18	1.04	0.9	0.83	1.00	1.17	0.84
66		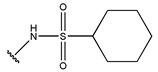			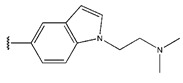		20	1.30	1.05	1.11	1.02	1.39	0.97	1.29
67 *		-NHSO_2_Et			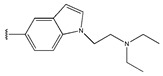		4.1	0.61	0.62	0.55	0.94	1.08	0.56	0.41
68		-NHSO_2_Et			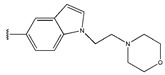		6.3	0.80	1.09	0.67	0.49	1.06	1.03	0.81
69 *		-NHSO_2_Et			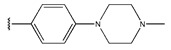		1.8	0.26	0.44	0.4	0.76	1.11	0.12	0.44
70 *		-NHSO_2_Et			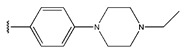		2.2	0.34	0.53	0.41	0.90	0.99	0.36	0.53
71		-NHSO_2_Et			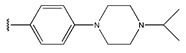		3.7	0.57	0.58	0.57	0.61	0.97	0.53	0.48
72		-NHSO_2_Et			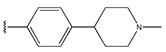		2.1	0.32	0.50	0.41	0.53	0.88	0.01	0.33
73		-NHSO_2_Et			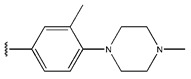		3.6	0.56	0.53	0.41	0.67	0.99	0.2	0.51
74 *		-NHSO_2_Et			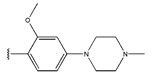		2.5	0.40	0.55	0.59	0.97	1.02	0.74	0.67
75		-NHSO_2_Et			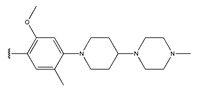		10	1.00	1.27	1.2	0.67	1.19	1.47	1.01
76 *		-NHSO_2_Et			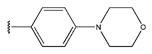		3.7	0.57	0.40	0.4	1.19	1.00	0.19	0.46
77 *		-NHSO_2_Et			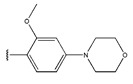		8.5	0.93	0.48	0.7	1.65	1.33	0.93	0.75
78		-NHSO_2_Et			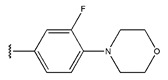		4.1	0.61	0.39	0.57	0.73	1.0	0.65	0.62
79		-NHSO_2_Et			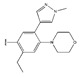		70.1	1.85	1.13	1.36	1.24	1.67	1.62	1.84
80		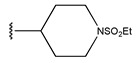			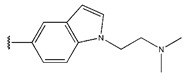		5.1	0.71	1.39	1.23	0.52	1.03	1.39	1.28
81		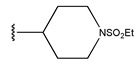			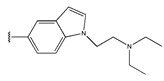		34	1.53	1.46	1.6	0.97	1.50	1.47	1.52
82 *		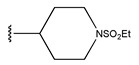			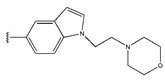		71	1.85	1.93	1.68	0.53	0.85	1.52	1.85
83		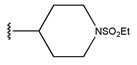			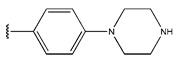		7.9	0.90	1.33	1.16	0.78	1.07	1.36	1.21
84		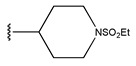			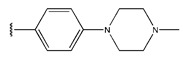		18	1.26	1.37	1.19	1.12	1.35	1.37	1.27
85		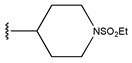			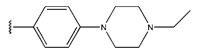		26	1.41	1.41	1.54	1.16	1.43	1.41	1.42
86		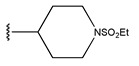			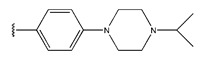		49	1.69	1.44	1.48	1.42	1.59	1.42	1.44
87		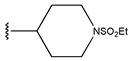			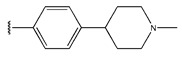		15	1.18	1.39	1.53	0.95	1.31	1.38	1.21
88 *		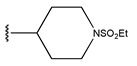			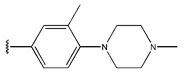		36	1.56	1.41	1.54	1.02	1.25	1.52	1.53
89		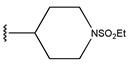			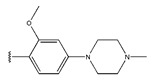		71	1.85	1.43	1.72	1.95	1.67	1.65	1.84
90		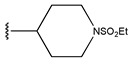			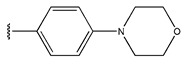		58	1.76	1.32	1.56	1.72	1.62	1.39	1.37
91		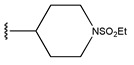			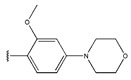		71	1.85	1.38	1.73	1.52	1.67	1.73	1.84
92		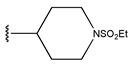			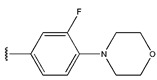		77	1.89	1.31	1.58	1.82	1.70	1.38	1.21

* test set.

## Data Availability

Data is contained within the article.

## References

[1] Sung H., Ferlay J., Siegel R.L., Laversanne M., Soerjomataram I., Jemal A., Bray F. (2021). Global cancer statistics 2020: GLOBOCAN estimates of incidence and mortality worldwide for 36 cancers in 185 countries. CA A Cancer J. Clin..

[2] World Health Organization: WHO (2023). Lung Cancer. https://www.who.int/news-room/fact-sheets/detail/lung-cancer.

[3] Shah R., Lester J.F. (2020). Tyrosine kinase inhibitors for the treatment of EGFR mutation-positive non–small-cell lung cancer: A clash of the generations. Clin. Lung Cancer.

[4] Paez J.G., Janne P.A., Lee J.C., Tracy S., Greulich H., Gabriel S., Herman P., Kaye F.J., Lindeman N., Boggon T.J. (2004). EGFR mutations in lung cancer: Correlation with clinical response to gefitinib therapy. Science.

[5] Dowell J., Minna J.D., Kirkpatrick P. (2005). Erlotinib hydrochloride. Nat. Rev. Drug Discov..

[6] Wu Y.L., Cheng Y., Zhou X., Lee K.H., Nakagawa K., Niho S., Tsuji F., Linke R., Rosell R., Corral J. (2017). Dacomitinib versus gefitinib as first-line treatment for patients with EGFR-mutation-positive non-small-cell lung cancer (ARCHER 1050): A randomised, open-label, phase 3 trial. Lancet Oncol..

[7] Nelson V., Ziehr J., Agulnik M., Johnson M. (2013). Afatinib: Emerging next-generation tyrosine kinase inhibitor for NSCLC. OncoTargets Ther..

[8] Kobayashi S., Boggon T.J., Dayaram T., Jänne P.A., Kocher O., Meyerson M., Johnson B.E., Eck M.J., Tenen D.G., Halmos B. (2005). EGFR mutation and resistance of non–small-cell lung cancer to gefitinib. New Engl. J. Med..

[9] Zhou W., Ercan D., Chen L., Yun C.H., Li D., Capelletti M., Cortot A.B., Chirieac L., Iacob R.E., Padera R. (2009). Novel mutant-selective EGFR kinase inhibitors against EGFR T790M. Nature.

[10] Ricciuti B., Baglivo S., Paglialunga L., De Giglio A., Bellezza G., Chiari R., Crinò L., Metro G. (2017). Osimertinib in patients with advanced epidermal growth factor receptor T790M mutation-positive non-small cell lung cancer: Rationale, evidence and place in therapy. Ther. Adv. Med Oncol..

[11] Schmid S., Li J.J., Leighl N.B. (2020). Mechanisms of osimertinib resistance and emerging treatment options. Lung Cancer.

[12] Dong H., Ye X., Zhu Y., Shen H., Shen H., Chen W., Ji M., Zheng M., Wang K., Cai Z. (2023). Discovery of Potent and Wild-Type-Sparing Fourth-Generation EGFR Inhibitors for Treatment of Osimertinib-Resistance NSCLC. J. Med. Chem..

[13] Zhu Y., Ye X., Shen H., Li J., Cai Z., Min W., Hou Y., Dong H., Wu Y., Wang L. (2023). Discovery of Novel Fourth-Generation EGFR Inhibitors to Overcome C797S-Mediated Resistance. J. Med. Chem..

[14] Tropsha A. (2010). Best practices for QSAR model development, validation, and exploitation. Mol. Inform..

[15] Li J., Cheng K., Wang S., Morstatter F., Trevino R.P., Tang J., Liu H. (2017). Feature selection: A data perspective. ACM Comput. Surv. CSUR.

[16] Bader R.F. (1985). Atoms in molecules. Acc. Chem. Res..

[17] Lipkin M.R., Martin C.C. (1947). Calculation of Weight Per Cent Ring and Number of Rings per Molecule for Aromatics. Anal. Chem..

[18] Kwon Y. (2001). Handbook of Essential Pharmacokinetics, Pharmacodynamics and Drug Metabolism for Industrial Scientists.

[19] Ertl P., Rohde B., Selzer P. (2000). Fast calculation of molecular polar surface area as a sum of fragment-based contributions and its application to the prediction of drug transport properties. J. Med. Chem..

[20] Smith G.F. (2011). Designing drugs to avoid toxicity. Prog. Med. Chem..

[21] Eriksson L., Jaworska J., Worth A.P., Cronin M.T., McDowell R.M., Gramatica P. (2003). Methods for reliability and uncertainty assessment and for applicability evaluations of classification-and regression-based QSARs. Environ. Health Perspect..

[22] Sosnowska A., Grzonkowska M., Puzyn T. (2017). Global versus local QSAR models for predicting ionic liquids toxicity against IPC-81 leukemia rat cell line: The predictive ability. J. Mol. Liq..

[23] (1994). Hyperchem.

[24] Loomis R.A., McGuire B.A., Shingledecker C., Johnson C.H., Blair S., Robertson A., Remijan A.J. (2015). Investigating the minimum energy principle in searches for new molecular species—The case of H2C3O isomers. Astrophys. J..

[25] Stewart J.J. (1990). MOPAC: A semiempirical molecular orbital program. J. Comput.-Aided Mol. Des..

[26] Katritzky A.R., Kulshyn O.V., Stoyanova-Slavova I., Dobchev D.A., Kuanar M., Fara D.C., Karelson M. (2006). Antimalarial activity: A QSAR modeling using CODESSA PRO software. Bioorganic Med. Chem..

[27] Gao Z., Xia R., Zhang P. (2022). Prediction of anti-proliferation effect of [1, 2, 3] triazolo [4, 5-d] pyrimidine derivatives by random forest and mix-kernel function SVM with PSO. Chem. Pharm. Bull..

[28] Wang Y., Liu Z., Qu A., Zhang P., Si H., Zhai H. (2020). Study of Tacrine Derivatives for Acetylcholinesterase Inhibitors Based on Artificial Intelligence. Lat. Am. J. Pharm..

[29] LI G., Wang X., LI A., Zhang P. (2023). QSAR Study on the IC_50_ of Thiosemicarbazone Derivatives as PC-3 Inhibitors Based on Mixed Kernel Function Support Vector Machine. Lat. Am. J. Pharm..

[30] Wang Y., Zhang P. (2023). Prediction of histone deacetylase inhibition by triazole compounds based on artificial intelligence. Front. Pharmacol..

[31] Hira Z.M., Gillies D.F. (2015). A review of feature selection and feature extraction methods applied on microarray data. Adv. Bioinform..

[32] Yang X., Qiu H., Zhang Y., Zhang P. (2023). Quantitative Structure-Activity Relationship Study of Amide Derivatives as Xanthine Oxidase Inhibitors using machine learning. Front. Pharmacol..

[33] Breiman L. (2017). Classification and Regression Trees.

[34] Zheng H., Yuan J., Chen L. (2017). Short-term load forecasting using EMD-LSTM neural networks with a Xgboost algorithm for feature importance evaluation. Energies.

[35] Biau G., Scornet E. (2016). A random forest guided tour. Test.

[36] Rigatti S.J. (2017). Random forest. J. Insur. Med..

[37] Ali J., Khan R., Ahmad N., Maqsood I. (2012). Random forests and decision trees. Int. J. Comput. Sci. Issues.

[38] Ferreira C. (2001). Gene expression programming: A new adaptive algorithm for solving problems. arXiv.

[39] Ferreira C. (2002). Gene expression programming in problem solving. Soft Computing and Industry: Recent Applications.

[40] Zhang C., Zhang Y., Shi X., Almpanidis G., Fan G., Shen X. (2019). On incremental learning for gradient boosting decision trees. Neural Process. Lett..

[41] Sun R., Wang G., Zhang W., Hsu L.T., Ochieng W.Y. (2020). A gradient boosting decision tree based GPS signal reception classification algorithm. Appl. Soft Comput..

[42] Cortes C., Vapnik V. (1995). Support-vector networks. Mach. Learn..

[43] Chen Y., Xu P., Chu Y., Li W., Wu Y., Ni L., Bao Y., Wang K. (2017). Short-term electrical load forecasting using the Support Vector Regression (SVR) model to calculate the demand response baseline for office buildings. Appl. Energy.

[44] Cyganek B., Krawczyk B., Woźniak M. (2015). Multidimensional data classification with chordal distance based kernel and support vector machines. Eng. Appl. Artif. Intell..

[45] Fathi Hafshejani S., Moaberfard Z. (2023). A new trigonometric kernel function for support vector machine. Iran J. Comput. Sci..

[46] Chen D.G., Wang H.Y., Tsang E.C. Generalized Mercer theorem and its application to feature space related to indefinite kernels. Proceedings of the 2008 International Conference on Machine Learning and Cybernetics.

[47] Hu M., Chen Y., Kwok J.T.Y. (2009). Building sparse multiple-kernel SVM classifiers. IEEE Trans. Neural Netw..

[48] Liang J.J., Qin A.K., Suganthan P.N., Baskar S. (2006). Comprehensive learning particle swarm optimizer for global optimization of multimodal functions. IEEE Trans. Evol. Comput..

[49] Liang J.J., Qin A.K., Suganthan P.M., Baskar S. Particle swarm optimization algorithms with novel learning strategies. Proceedings of the 2004 IEEE International Conference on Systems, Man and Cybernetics (IEEE Cat. No. 04CH37583).

[50] Nagelkerke N.J. (1991). A note on a general definition of the coefficient of determination. Biometrika.

[51] Chicco D., Warrens M.J., Jurman G. (2021). The coefficient of determination R-squared is more informative than SMAPE, MAE, MAPE, MSE and RMSE in regression analysis evaluation. PeerJ Comput. Sci..

[52] Chirico N., Gramatica P. (2012). Real external predictivity of QSAR models. Part 2. New intercomparable thresholds for different validation criteria and the need for scatter plot inspection. J. Chem. Inf. Model..

[53] Sahigara F., Mansouri K., Ballabio D., Mauri A., Consonni V., Todeschini R. (2012). Comparison of different approaches to define the applicability domain of QSAR models. Molecules.

[54] Tropsha A., Gramatica P., Gombar V.K. (2003). The importance of being earnest: Validation is the absolute essential for successful application and interpretation of QSPR models. QSAR Comb. Sci..

[55] Di L., Kerns E.H., Carter G.T. (2009). Drug-like property concepts in pharmaceutical design. Curr. Pharm. Des..

[56] Morris G.M., Lim-Wilby M. (2008). Molecular docking. Methods Mol. Biol..

